# Methods for Comprehensive Calibration of a Low-Frequency Angular Acceleration Rotary Table

**DOI:** 10.3390/s23104876

**Published:** 2023-05-18

**Authors:** Renjian Feng, Jiaxuan Yan, Yinfeng Wu, Ning Yu, Xudong Yin

**Affiliations:** 1 Key Laboratory of Education Ministry for Precision Opto-Mechatronics Technology, Beijing 100191, China; 2 School of Instrumentation and Optoelectronic Engineering, Beihang University, Beijing 100191, China

**Keywords:** fiber optical gyroscope, optical shaft encoder, rotary table calibration, total harmonic distortion

## Abstract

The total harmonic distortion (THD) index and its calculation methods are presented to calibrate the sinusoidal motion of the low-frequency angular acceleration rotary table (LFAART) and make up the incomprehensive evaluation based on the angular acceleration amplitude and frequency error indexes. The THD is calculated from two measurement schemes: a unique scheme combining the optical shaft encoder and the laser triangulation sensor and a regular scheme using the fiber optical gyroscope (FOG). An improved reversing moments recognition method is presented to upgrade the accuracy of solving the angular motion amplitude based on optical shaft encoder output. The field experiment shows that the difference in the THD values achieved using the combining scheme and FOG is within 0.11% when the signal-to-noise ratio of the FOG signal is higher than 7.7 dB, indicating the accuracy of the proposed methods and the feasibility of taking THD as the index.

## 1. Introduction

A rotary table is a standard source that can be set manually to produce the desired angular motion [1]. It can reproduce dynamic angle values and is used mainly to test the performance of inertial navigation systems and inertial devices [2,3,4]. A rotary table usually outputs the preset angle, angular velocity, and angular acceleration. However, there is a non-ideal movement of the rotary table during the actual calibration, which directly impacts the evaluation of the inertial devices and further affects their performance. Thus, the quantitative description of angular motion parameters of the rotary table is of great significance. From a metrological point of view, the calibration can help realize the traceability from the angular motion to the angular standard. Proceeding from the actual, it helps improve the rotary table’s performance. Moreover, it accounts for the non-ideality of the rotary table movement in the calibration of the inertial devices so that the corresponding compensation can be calculated to improve the calibration accuracy. The rotary table calibration precisely aims to describe their non-ideal motion quantitatively and realize the traceability of angular motion parameters.

There is a plethora of research on the static calibration of the rotary table, including the calibration of the angle positioning error and motion error of other degrees of freedom, such as end face run-out and axial shaking [5,6]. However, there is little research on dynamic calibration. Various carriers with inertial devices possess high maneuverability in the actual movement, so the motion of the rotary table should not be limited to rotational positioning and uniform motion. Sinusoidal rotation is a kind of motion in which angular displacement, angular velocity, and angular acceleration change sinusoidally with time, reflecting the dynamic characteristics of the rotary table thoroughly. Moreover, the sinusoidal motion of the rotary table is the calibration object considered in this work. Different from the standard vibrators described in the national metrological verification Regulation of China JJG 298-2015 [7] and the angular vibration table in ISO 16063-15 [8], the rotary table with sinusoidal motion in this paper has a higher rotation amplitude and lower frequency, called low-frequency angular acceleration rotary table (LFAART). Because the angular vibration table only produces high-frequency and small-amplitude sinusoidal motion, excluding low-frequency and large-amplitude motion, its calibration for inertial devices is limited. Fortunately, the LFAART makes up for the limitation through its specific movement and can be used to test any gyroscope when its angular velocity and acceleration match that of the table. Thus, the calibration of LFAART is essential to the comprehensive evaluation of inertial devices. Because of the large amplitude of LFAART’s motion, calibration methods for the angular vibration table are no longer applicable, mainly due to their poor measurement range. Moreover, there is little research on LFAART and its calibration. Therefore, the calibration of LFAART is a critical problem that is difficult to solve.

The rotary table is generally considered as a standard source that generates the sinusoidal angular motion to test the dynamic specifications of inertia device or system. Thus, the movement is treated as an ideal sinusoidal form, and only amplitude and frequency are concerned. In [9], an optical shaft encoder (OSE) and a laser triangulation sensor (LTS) was used to measure the amplitude and frequency of the angular acceleration. The OSE signal series is recorded when the swing amplitude is large enough. Then the motion cycle is recognized according to the zero-crossings of the series, and angular displacement within the cycle is calculated by counting the fringe. The LTS method is applied for the small angular amplitude. The motion of the rotary table is not flawlessly sinusoidal. The evaluation method of LFAART only includes the angular acceleration amplitude, and the frequency index is not comprehensive.

Total harmonic distortion (THD) is initially used to evaluate the quality of electrical signals. According to the standard IEEE 1057-2017, THD is defined as follows [10]:

(1) $$THD=\sqrt{\frac{\sum_{h=2}^{N_{H}}A_{h}{}^{2}}{A_{1}{}^{2}}}\times 100\%$$

where $N_{H}$ denotes the highest order of the harmonic component, and the typical value is 10, $A_{h}$ is the amplitude of the *h*th order harmonic, and $A_{1}$ is the amplitude of the first harmonic. Moreover, the IEEE 1057-2017 standard also recommends three methods to calculate the amplitude of each harmonic and includes detailed calculation formulae. The sinusoidal fitting method has higher computational accuracy and better robustness than the other methods.

This paper studies the calibration method for LFAART based on the THD index. This purpose uses two measurement schemes, a unique measurement scheme that combines the optical shaft encoder and the laser triangulation sensor and a regular scheme using the fiber optical gyroscope (FOG). For the current measurement scheme, the proposed work focuses on the data processing of the OSE output because the output signal of the LTS is simple. Under the actual motion with stagnation, backwardness, and creep, the phase modulation signal of optical shaft encoder output is complex to solve. The proposed improved method can solve the signal more accurately than the existing method. For FOG, a method is proposed for calculating the THD. Moreover, the experiment is designed and implemented to prove the correctness of the proposed procedures and the feasibility of using THD as an evaluation index for the LFAART’s sinusoidal motion.

## 2. The Indispensability of Applying THD as an Index

This chapter explains the importance of bringing the THD as an index through comparisons with other evaluation indexes. Because there is little research about the calibration of rotary tables with sinusoidal motion, we extend the comparison scope to the rotary table evaluation indexes, namely, not specifying the sinusoidal motion. Moreover, we just focus on the dynamic calibration indexes because the static calibration has been thoroughly studied and is not our point. The comparisons are listed as follows.

### 2.1. Angular Acceleration Amplitude Error and Angular Motion Frequency Error

**Figure 1:**
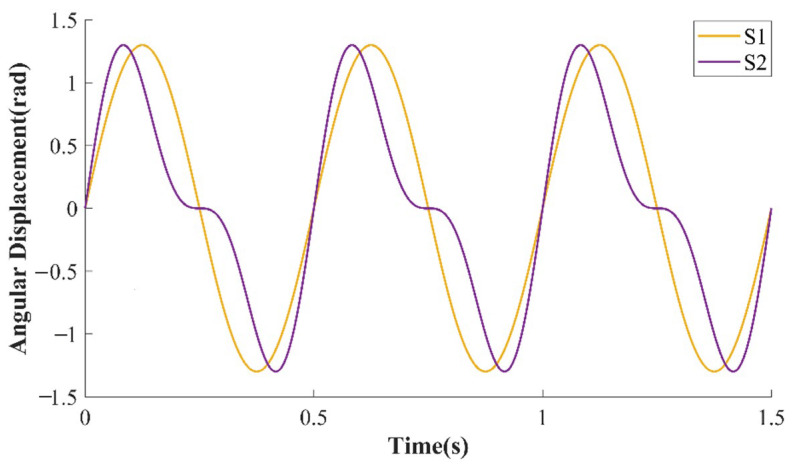
Time-dependent curves of angular displacement with ideal and distorted motion.

Angular acceleration amplitude and motion frequency errors are used as calibration items in [9] and China standardization JJF 1210-2008—Calibration Specification for Rate Table [11]. As shown in Figure 1, $S_{1}$ and $S_{2}$ both are curves of angular displacement of the LFAART varied with time and $S_{1}$ is an ideal sinusoidal curve. Although the maximum angular acceleration amplitude and frequency of $S_{1}$ and $S_{2}$ are the same, their angular motion is quite different. Total harmonic distortion (THD) can quantify the difference. For example, the THD of $S_{1}$ is 0% while $S_{2}$ is 25%. Thus, adding the THD as an index can comprehensively reflect the non-ideality of LFAART’s sinusoidal motion.

### 2.2. Angular Position Error

Angular position error is also shown as the calibration results in the studies [5,12,13,14]. The form of the results can be an angle-dependent curve of error or the max absolute value of the error. Although those previous research always apply angular position error as an index to evaluate the rotary table with uniform motion, we can easily deduce the angular position error in the sinusoidal motion. By taking the sinusoidal curve with setting amplitude and frequency as the ideal motion and comparing it against the measured angular displacement, the error of each measurement point is given as follows:

(2) $$ap_{err}\left(t_{i}\right)=ap_{meas}\left(t_{i}\right)-ap_{ideal}\left(t_{i}\right)$$

where $ap_{meas}\left(t_{i}\right)$ is the measurement value at time epoch $t_{i}$, $ap_{ideal}$ is the ideal value, and $ap_{err}$ is the angular position error.

Angular position error is not a perfect index for the sinusoidal motion of the rotary table for the following reasons. First, it includes angular acceleration amplitude error and angular motion frequency error, leading to poor intuitiveness and analysis difficulty. Moreover, deciding if a rotary table meets the precision requirements through an error graph is a dilemma because there is no specific threshold value. In comparison, the max absolute value of the error indicates the feature of one typical point, which is uncomprehensive.

## 3. THD Calculation Method Adopting OSE

The OSE is widely used in angular motion parameters measurement due to its high accuracy, good dynamic characteristics, and wide measurement range [15,16]. The output signals of an OSE are two orthogonal phase-modulated sinusoidal signals with the same amplitude. The two signals’ phases are proportional to the measured object’s angular displacement, and the signals’ instantaneous frequency is proportional to the instantaneous angular rate. However, determining the rotation direction needs the phase comparison of the two signals. Based on the feature of the OSE signal, a recognition algorithm is proposed for LFAART’s rotation reversing moments in [9], i.e., moving backwards near the zero speed position. First, the signal period is divided into eight subintervals. Second, subintervals containing reversing moments are determined. Finally, the midpoints of these subintervals are taken as the estimates of reversing moments [9].

**Figure 2:**
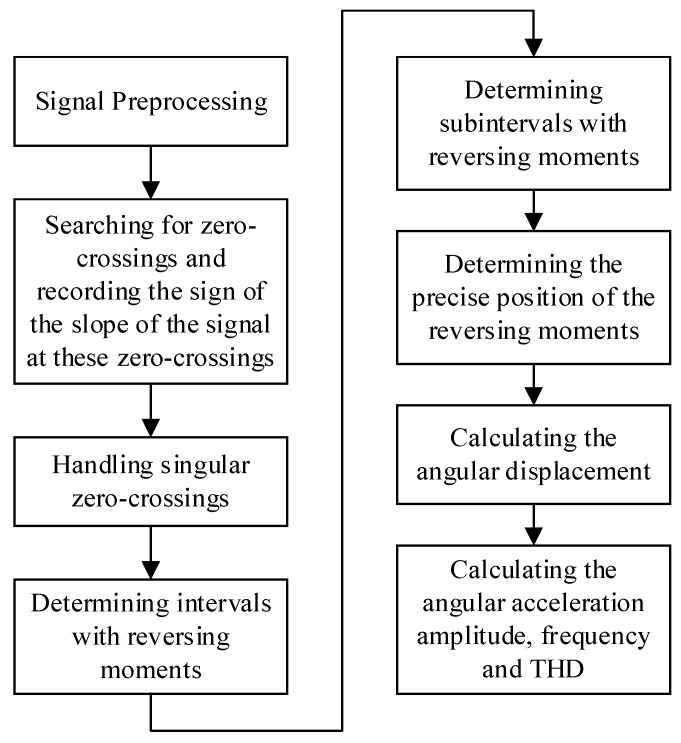
Flowchart of the THD calculation method based on OSE.

This paper has improved the earlier method, and the reversing moments have been estimated accurately to specific data points. The improved algorithm contains eight steps, as shown in Figure 2. The first four steps and the angular acceleration amplitude and frequency calculation method are similar to [9]. Thus, the improved algorithm is mainly described in the following part, including determining subintervals with reversing moments, determining the precise position of the reversing moments, calculating the angular displacement, and calculating the THD.

### 3.1. Determining Subintervals with Reversing Moments

Subdivision of the OSE signal is the precondition for determining subintervals with reversing moments. The traditional high subdivision methods mainly include arctangent, amplitude linearization, and closed-loop tracking. Ye et al. [17] proposed an accurate linearization subdivision method based on the traditional ones, reducing the calculation and ensuring subdivision accuracy. In this paper, every period of the OSE signal is divided into twelve subintervals based on the method given by Ye et al. [17]. When the rotation direction $Sdir_{k}$ of LFAART equals 1, subintervals decrease cyclicly along the time axis as follows:

(3) {12,11,10,9,8,7,6,5,4,3,2,1,12,11,10,9…}

When *Sdir_k_* = −1, subintervals increase cyclicly along the time axis as follows:

(4) {1,2,3,4,5,6,7,8,9,10,11,12,1,2,3,4…}

The subintervals change the trend mainly because of LFAART’s reversion and LFAART’s non-ideal motion like stagnation, backwardness, creep, or the relatively low signal-to-noise ratio (SNR). The number of subintervals with continuous changes in the observed trend has been recorded to avoid the influence of these non-ideal factors on the recognition of reversing moments. No reversing moment is included in these subintervals when the number is even. When the number is odd, the medium subinterval is identified as the subinterval with reversing moment. For example, refer to (5); the number of times of continuous change in the subintervals’ trend is two. Thus, there is no reversing moment in these subintervals.

(5) {2,3,4,5,4,5,6,7,8,…}

When the subintervals come with (6), the number is three. Moreover, the second “7” is accepted as the subinterval with reversing moment.

(6) {4,5,6,7,8,7,8,7,6,5…}

### 3.2. Determining the Precise Position of the Reversing Moments

**Figure 3:**
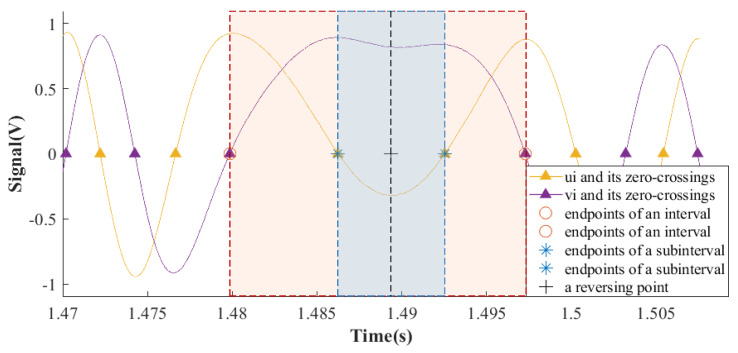
Example of an interval, a subinterval, and a reversing point.

Figure 3 is an example of an interval, a subinterval, and a reversing point. The red region is an interval; the blue part is a subinterval, and the mark “+” is a reversing moment. In Section 3.1, the determination of a subinterval like the blue part in Figure 3 has been elaborated. This part explains obtaining a specific data point like the mark “+” in Figure 3. Firstly, one of the two signals output by OSE for subsequent calculation is selected according to the intensity of change in the subinterval. Two signals output by OSE after sampling and preprocessing are named as $u_{i}$ and *v_i_*, respectively, where i is a positive integer, indicating the index of discrete signals. Let the indexes to the left and right endpoints of the subinterval be $Sub^{-}$ and $Sub^{+}$. The changing intensity of $u_{i}$ in the subinterval is quantified as *qu_jud_*, where

(7) $$qu_{jud}=\overset{Sub^{+}}{\underset{j=Sub^{-}}{\sum }}\left(u_{j}-\frac{u_{Sub^{-}}+u_{Sub^{+}}}{2}\right)$$

The changing intensity of $v_{i}$ in the subinterval is quantified as $qv_{jud}$, where

(8) $$qv_{jud}=\overset{Sub^{+}}{\underset{j=Sub^{-}}{\sum }}\left(v_{j}-\frac{v_{Sub^{-}}+v_{Sub^{+}}}{2}\right)$$

When $\left|qu_{jud}\right|>\left|qv_{jud}\right|$, $u_{i}$ changes rapidly. Therefore, $u_{i}$ is used for subsequent calculation. When $\left|qu_{jud}\right|<\left|qv_{jud}\right|$, $v_{i}$ is chosen. In Figure 3, $\left|qu_{jud}\right|>\left|qv_{jud}\right|$, thus $u_{i}$ is chosen. Taking the signal $u_{i}$ as an example, when $qu_{jud}<0$, index *Chp* meets the condition: $\forall \;k\in \left[Sub^{-},\;Sub^{+}\right]$, s.t. $u_{Chp}\leq u_{k}$. When $qu_{jud}>0$, Chp meets the condition: $\forall \;k\in \left[Sub^{-},\;Sub^{+}\right]$, s.t. $u_{Chp}\geq u_{k}$. And $u_{Chp}$ is picked as the reversing point and Chp as the reversing moment.

### 3.3. Calculating the Angular Displacement and THD

We calculate the angular displacement $\theta_{i}$ corresponding to all data points based on the method proposed by Ye et al. To ensure the calculation accuracy of THD, the angular displacement $\theta_{i}$ of several full sinusoidal rotation periods should be taken for the calculation. The interception relies on the reversing moments’ recognition method proposed in Section 3.1 and Section 3.2. To be specific, when n motion periods are considered, the intercepted $\theta_{i}$ exactly includes 2n+1 reversing moments. Taking $\theta_{i}$ after intercepting the analysis object, the THD of the angular displacement is calculated based on the sine-fitting method according to IEEE standard 1057-2017.

## 4. THD Calculation Method Adopting FOG

The FOG is widely used in angular velocity measurement because of its small volume, high reliability, and high interference immunity to acceleration. The angular velocity of the carrier can be measured directly because FOG’s output signal is proportional to the sensitive axis’s angular velocity. However, noise is generated in almost all FOG parts, including fiber coil, light source, detector, coupler, phase modulator, etc., which leads to low SNR [18]. Therefore, filtering estimation methods [18,19,20] generally must be applied to solve the angular velocity. Moreover, the angular velocity measured by FOG must be integrated to obtain angular displacement, bringing inevitable accumulation error. A FOG-based THD calculation method is proposed to address the above two problems. This method implements sine fitting on the measurement data of FOG directly and calculates the THD based on the integration relationship between angular velocity and angular displacement. This method makes full use of the robustness of sine fitting, which avoids the complicated processing for noise reduction and drift reduction and ensures high-precision calculation of THD.

Supposing the fitting function $\dot{\theta }\left(t\right)$ of the angular velocity of LFAART after sine fitting is shown in the following equation:

(9) $$\dot{\theta }\left(t\right)=D_{0}+B_{1}\cos\left(2\pi f_{1}t\right)+\ldots +B_{\;N_{H}}\cos\left(2\pi f_{\;N_{H}}t\right)\;+C_{1}\sin\left(2\pi f_{1}t\right)+\ldots +C_{\;N_{H}}\sin\left(2\pi f_{\;N_{H}}t\right)$$

where $D_{0},\;B_{1}\ldots B_{\;N_{H}},\;C_{1}\ldots C_{\;N_{H}},\;f_{1}\ldots f_{\;N_{H}}$ are parameters solved by the sine fitting method. Let $A_{h}{}^{\prime }=\sqrt{B_{h}{}^{2}+C_{h}{}^{2}}$, $\tan\varphi_{h}=B_{h}/C_{h}$, then (9) can be rewritten as

(10) $$\dot{\theta }\left(t\right)=D_{0}+A_{1}{}^{\prime }sin\left(2\pi f_{1}t+\varphi_{1}\right)+\ldots +A_{N_{H}}{}^{\prime }sin\left(2\pi f_{N_{H}}t+\varphi_{N_{H}}\right)$$

According to the relationship between angular velocity $\dot{\theta }\left(t\right)$ and angular displacement θ(t), also known as

(11) $$\theta \left(t\right)=\int_{0}^{t}\dot{\theta }\left(x\right)dx$$

the fitting function θ(t) of the angular displacement of LFAART is

(12) $$\theta \left(t\right)=D_{0}t-\frac{A_{1}{}^{\prime }}{2\pi f_{1}}\cos\left(2\pi f_{1}t+\varphi_{1}\right)+\frac{A_{1}{}^{\prime }}{2\pi f_{1}}\cos\varphi_{1}-\ldots \;-\frac{A_{\;N_{H}}{}^{\prime }}{2\pi f_{\;N_{H}}}\cos\left(2\pi f_{\;N_{H}}t+\varphi_{N_{H}}\right)+\frac{A_{\;N_{H}}{}^{\prime }}{2\pi f_{\;N_{H}}}\cos\varphi_{N_{H}}$$

Assuming $D=D_{0}t+\frac{A_{1}{}^{\prime }}{2\pi f_{1}}\cos\varphi_{1}+\ldots +\frac{A_{\;N_{H}}{}^{\prime }}{2\pi f_{\;N_{H}}}\cos\varphi_{N_{H}}$, thus θ(t) is

(13) $$\theta \left(t\right)=D-\frac{A_{1}{}^{\prime }}{2\pi f_{1}}\cos\left(2\pi f_{1}t+\varphi_{1}\right)-\ldots -\frac{A_{\;N_{H}}{}^{\prime }}{2\pi f_{\;N_{H}}}\cos\left(2\pi f_{\;N_{H}}t+\varphi_{N_{H}}\right)$$

Based on (1), the angular displacement $THD_{\theta }$ of LFAART is

(14) $$THD_{\theta }=\sqrt{\frac{\sum_{h=2}^{N_{H}}{\left(\frac{A_{h}{}^{\prime }}{2\pi f_{h}}\right)}^{2}}{{\left(\frac{A_{1}{}^{\prime }}{2\pi f_{1}}\right)}^{2}}}$$

## 5. Experiment

**Figure 4:**
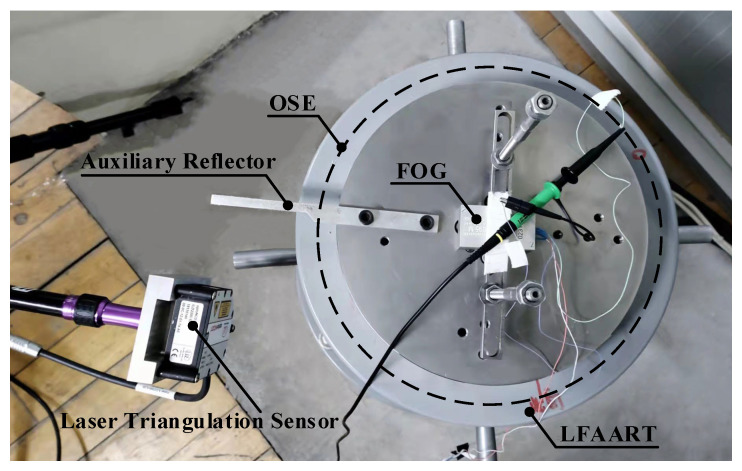
Picture of the field experiment.

In the experimental setup, OSE and LTS have been combined to measure the angular acceleration amplitude and frequency of LFAART. The line count of the OSE, HEIDeNHAIN RON886, is 36,000. An LTS with the model MICRO-EPSILON ILD 2300-2 is utilized. Its range and resolution are 2 mm and 0.03 mm, respectively. The model of the FOG is VG095M with a range of 0–300 °/s. The maximum frequency of the table is 10 Hz, and the angular motion amplitude is 5°. Moreover, the signal of FOG is simultaneously sampled to verify the scheme’s feasibility and the correctness of the data processing method. A picture of the field experiment is shown in Figure 4.

When the sinusoidal rotation amplitude is larger, the OSE is adopted, and the THD is calculated based on the method proposed in Section 3; when the amplitude is smaller, the LTS is utilized. The LTS measures the linear displacement of the auxiliary reflector, which is a specially designed workpiece screwed on the LFAART. According to the triangular relation, the angular displacement $\theta_{i}$ is

(15) $$\theta_{i}=\arctan\frac{D_{i}}{R}$$

where $D_{i}$ is the linear displacement measured by LTS, R is the distance from the laser spot to the center of the table. Because the signal of LTS is proportional to the object’s displacement, the complicated data processing procedure is not necessary, and the reversing moments can be simply gained by extreme value calculations. 

For comparison, we sample the data of FOG simultaneously, and the THD is solved on the method proposed in Section 4. The results of the experiment are listed in Table 1.

**Figure 5:**
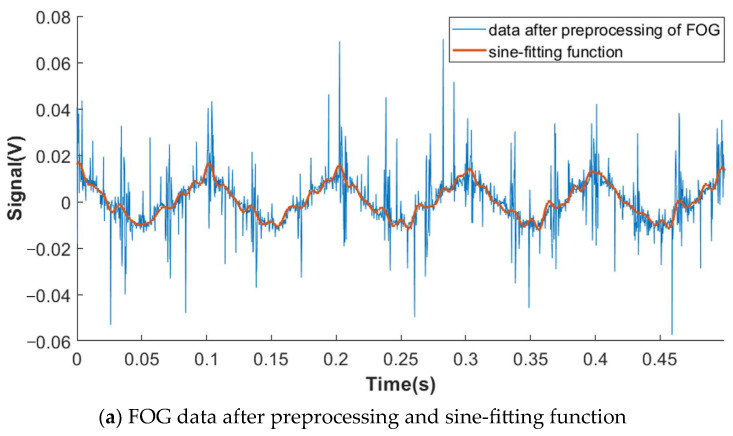

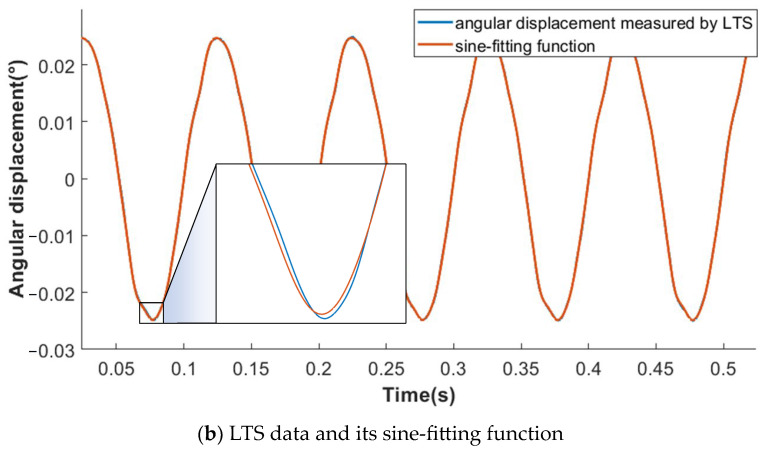
Sine fitting results of data of FOG and LTS with sinusoidal angular motion (amplitude: 0.025°; frequency: 10 Hz).

As shown in Table 1, the difference value between THD calculated by the proposed two-measurement scheme is within 0.11% except for the first experiment, validating the method proposed in Section 3 and Section 4. With an angular motion amplitude of 0.025° and a frequency of 10 Hz, the difference value between FOG and LTS is unacceptably huge. This is because the angular velocity of LFAART is relatively small, and then the SNR is quite low. So, the sine-fitting result is greatly influenced by serious noise, as shown in Figure 5a. Thus, comparing the results in such a situation is inappropriate, and the difference calculation does not make sense. However, from another dimension, we can conclude from Figure 5b that the sine-fitting function is close to the angular displacement measured by LTS, and the root mean square of their difference is 0.0001, indicating the credibility of the THD calculation result based on LTS.

**Figure 6:**
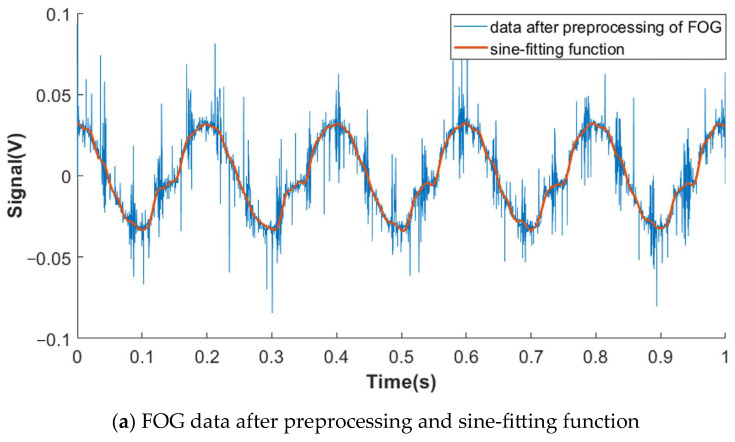

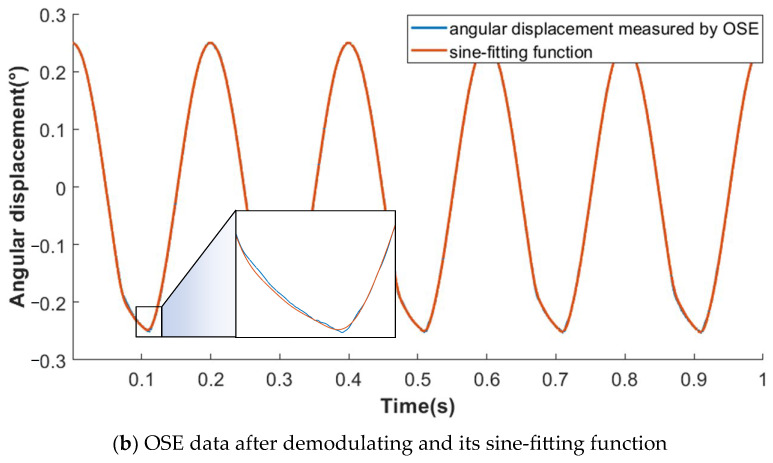
Sine fitting results of data of FOG and OSE with sinusoidal angular motion (amplitude: 0.1°; frequency: 5 Hz).

The difference value is just 0.11% with an angular motion amplitude of 0.1° and a frequency of 5 Hz. Moreover, the FOG data (after preprocessing) and sine fitting function are shown in Figure 6a. The OSE data after demodulating and its sine fitting function are shown in Figure 6b. Both gain a well-fitting effect. Thus, the measurement results of these two sensors are reciprocal and supportive.

Under this circumstance, the SNR is 7.7 dB by taking the fitting function as the clean signal and the difference value between the measurement data and the fitting function as the noise. Therefore, we can summarize that the THD can be solved precisely based on FOG when the SNR is higher than 7.7 dB, while based on our combined scheme, the THD calculation is always accurate.

## 6. Conclusions

In this paper, THD has been proposed as an index of the sinusoidal motion evaluation of LFAART and calculation methods of THD based on the combining scheme and FOG, respectively. The field experiment shows that the difference value between the combining scheme and FOG is within 0.11% when the SNR of the FOG signal is higher than 7.7 dB, indicating the accuracy of the proposed methods. The THD index and the corresponding method proposed in the paper remarkably improve the comprehensiveness of the LFAART calibration. The proposed work has a significant practical value and can also be used to evaluate and calibrate other inertial test devices’ sinusoidal motion. However, we mainly focus on the distortion measurement of the sinusoidal motion of the LFAART. The THD value has not been adopted yet to optimize the table’s control strategy. Calibration schemes and methods with traceability and high accuracy will be studied in the future for the rotary table with higher dynamic and random movement.

## Figures and Tables

**Table 1:** THD calculation results are based on OSE, LTS, and FOG under different sinusoidal rotation amplitude and frequency.

Angular Motion Amplitude (°)	Angular Motion Frequency (Hz)	THD Calculation Results (%)			Difference Value of THD (%)
		Based on the Combined Measurement Scheme		Based on FOG	
		Based on OSE	Based on LTS		
0.025	10	──	4.49	7.50	−3.01
0.1	5	5.42	──	5.31	0.11
0.25	3	2.79	──	2.81	−0.02
0.5	2	1.38	──	1.41	−0.03
5	0.5	0.21	──	0.21	0

## Data Availability

The data presented in this study are available on request from the corresponding author.
